# Analysis of genetic alterations identifies the frequent mutation of *GNAS* in colorectal laterally spreading tumors

**DOI:** 10.1002/cac2.12085

**Published:** 2020-08-06

**Authors:** Yanying Nong, Yuru Zhang, Yi Zhang, Lei Pan, Junsheng Chen, Chaojun Zhu, Lu Han, Aimin Li, Side Liu

**Affiliations:** ^1^ Guangdong Provincial Key Laboratory of Gastroenterology Department of Gastroenterology Nanfang Hospital Southern Medical University Guangzhou Guangdong 510515 P. R. China

**Abbreviations**
ACVR1B: activin A receptor type 1B
ACVR2A: activin A receptor type 2A
APC: adenomatous polyposis coli
ASPM: abnormal spindle microtubule assembly
BMPR1A: bone morphogenetic protein receptor type 1A
BRAF: V‐raf murine sarcoma viral oncogene homolog B
cAMP: cyclic adenosine monophosphate
COX‐2: clooxygenase‐2
CRC: colorectal cancer
CRN: colorectal neoplasm
CTNNB1: catenin β 1
EPB41L4A: erythrocyte membrane protein band 4.1 like 4A
FAT4: FAT atypical cadherin 4
FBXW7: F‐box and WD repeat domain containing 7
GLI1: glioma‐associated oncogene homolog 1
GNAS: G protein subunit α
HGIN: high‐grade intraepithelial neoplasia
indel: insertion/deletion
KRAS: V‐ki‐ras2 kirsten rat sarcoma 2 viral oncogene homolog
LGIN: low‐grade intraepithelial neoplasia
LST: laterally spreading tumor
MAPK: mitogen‐activated protein kinase
MED12L: mediator complex subunit 12L
MLH1: MutL homolog 1
NRXN1: neurexin 1
PIK3CA: phosphatidylinositol‐4,5‐bisphosphate 3‐kinase catalytic subunit alpha
Rap1: ras‐proximity 1
RIOK2: RIO kinase 2
SDK1: sidekick cell adhesion molecule 1
SLCO4C1: solute carrier organic anion transporter family member 4C1
SMAD: small mothers against decapentaplegic homolog
SMG: significantly mutated gene
SNV: somatic nucleotide variant
SOX9: sex‐determining region Y‐box 9
TA: tubular adenoma
TGFB2: transforming growth factor β 2
TGF‐β: transforming growth factor‐β
TP53: tumor protein 53
TTN: titin
TVA: tubulovillous adenoma
VA: villous adenoma
WES: whole‐exome sequencing.

Dear Editor,

Colorectal cancer (CRC) is among the most commonly diagnosed cancer and the leading cause of cancer‐related death worldwide [1]. Most CRCs develop from adenomas. Laterally spreading tumors (LSTs) are non‐polypoid superficial colorectal neoplasms (CRNs) that are larger than 1 cm and typically extend laterally along the luminal wall. Based on surface morphology, it can be classified into a granular or nongranular type. The pathological morphology of LST manifests only as adenoma or with cancer. Recent studies have characterized 45‐79% of LSTs as high‐grade intraepithelial neoplasia (HGIN) or submucosal invasion; and the risk of submucosal invasion increases with lesion size [2]. Hence, LST is considered a precancerous CRC lesion. Patients with LSTs are usually asymptomatic and diagnosis typically occurs during physical examinations or screening colonoscopy. However, flat tumors are difficult to detect by colonoscopy and a missed LST may progress to invasive carcinoma within a few years [3]. Further, little is known about the etiopathogenetic mechanism of LST.

The “adenoma‐to‐carcinoma” pathogenesis involves complex molecular events associated with somatic mutations, but few studies have investigated such. A recent study examined the mutational profiles of 10 LSTs, but these were predominantly low‐grade intraepithelial neoplasia (LGIN) with very few recurrently mutated genes [4]. Thus, little is known about the genetic alterations in LST with HGIN.

Here, we performed whole‐exome sequencing (WES) of 14 LSTs with HGIN and their matched adjacent non‐tumor mucosa to identify possible somatic mutations. Sanger sequencing of 79 LSTs was then performed to validate the frequency of G protein subunit α (*GNAS*) p.R186C/R186H in LST.

Details of all procedures can be found in the Supplementary Materials. Briefly, for WES (mean depth: 118×), formalin‐fixed paraffin‐embedded tissues were micro‐dissected to separate the HGIN from the tumor and adjacent non‐tumor tissues. Sequencing was performed using the Illumina HiSeq X (Illumina Inc., San Diego, CA, USA). Somatic nucleotide variants (SNVs) and insertions/deletions (indels) were identified using MuTect2 [5]. Indels, nonsense SNVs, and the variants at the splicing sites were defined as inactive mutations.

The clinicopathological characteristics of the 14 investigated patients are presented in Table S1 and their endoscopic images in Fig. S1. The total number of mutations in each patient is presented in Table S2. Analysis of protein‐changing variants showed a median of 79 SNVs (mean ± standard deviation: 155 ± 259) and 17 indels (26 ± 27) (Table S3; Fig. S2A). The median mutation burden was 3.8/Mb (7.3 ± 12.5/Mb) (Table S2; Fig.S2B), which was greater than in previous reports of LST with LGIN [4]. A median of 93 genes were mutated (179 ± 266; Table S2). 202 genes were recurrently mutated and 38 genes had mutations in ≥ 20% of the samples (Figure 1A). Eight LSTs (57%) had *APC* mutations among 11 mutation sites, of which 8 were nonsense SNVs and 3 were indels (Figure 1B). Seven LSTs (50%) had *GNAS* mutations, of which 5 were p.R186C/R186H (Figure 1C). *FAT4*, *SDK1*, and *TTN* were also commonly mutated at a 35.7% frequency (5/14). *KRAS* mutations were detected in 21.4% (3/14) of LSTs, all of which were in the codon 12 or 13 with a C > T substitution. *BRAF* mutation was found in 3 LSTs (p.D594N, p.V600E, and p.P403fs). One mutation was found in *TP53* but none in *CTNNB1* or *PIK3CA*. The high frequencies of *APC* and *KRAS* mutations and low frequencies of *TP53*, *CTNNB1*, and *PIK3CA* mutations were reported in previous investigations [4, 6].

**FIGURE 1:**
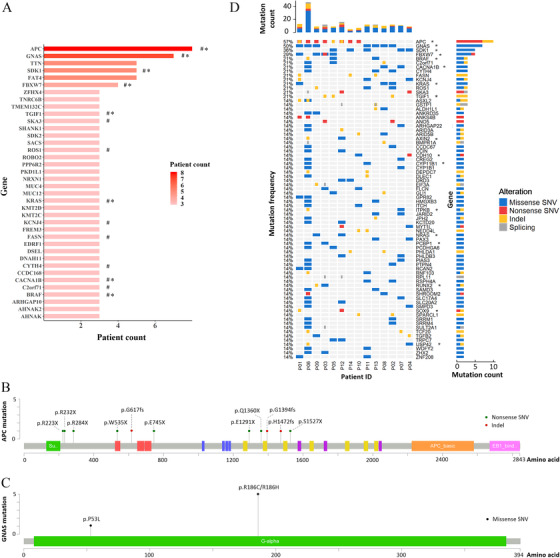
**Mutation frequency of subsets of genes and distribution of *APC* and *GNAS* mutations**. A. Genes with mutation frequencies greater than 20%; * the known driver genes, and ^#^ the SMGs. B. Lolliplot showing that the *APC* mutations were all inactive mutations with 8 nonsense SNVs and 3 indels. C. Lolliplot showing the *GNAS* mutational hotspot at p.R186C/R186H. The *GNAS* p.D583N in case P06 was not involved in G‐alpha protein. D. OncoPrint of recurrent SMGs. * the known driver genes. Mutations were classified as missense SNV, nonsense SNV, indel, or variant at splicing site. *APC*: adenomatous polyposis coli; *GNAS*: G protein subunit α, SMGs: significantly mutated genes, SNV: somatic nucleotide variant, indel: insertion/deletion

The present study is the first to report the frequent *GNAS* mutations in LST. The most frequently altered genes in our patients differed from those in a previous multi‐omics study of LST with LGIN [4]. Mutated genes such as *APC*, *NRXN1*, and *SOX9* were found in both LGIN and HGIN lesions. However, some of the recurrently altered genes, such as *MED12L*, *EPB41L4A*, *SLCO4C1*, *RIOK2*, and *ASPM*, of LGIN lesions were not detected in our cohort. This difference might be due to differences in the pathological severity of LST or in the methods used to identify altered genes. These results also suggest the involvement of varied key genes in different stages of LST development.

MutSigCV analysis [7] identified 368 significantly mutated genes (SMGs) (Table S4, Figure 1D). Previous research identified several of these genes (*APC*, *FBXW7*, *BRAF*, *ACVR2A*, *ACVR1B*, and *SOX9; ACVR2A* and *ACVR1B* were detected in only 1 case, therefore not shown in Figure 1D) as SMGs in CRC [8]. Among SMGs, 29 driver genes of colorectal adenocarcinoma were identified (Table S5).

Analysis of mutational signatures in 14 LSTs (Fig. S2C) indicated that signature 1A was present in all LSTs and had the greatest weight. Previous research reported that this signature had a strong relationship with age and was common in cancers [9]. One LST had signature 6 which stands out as many substitutions or small indels due to microsatellite instability from DNA mismatch repair deficiency [9]. This deficiency may be related to the increased methylation of *MLH1* in LST [6]. Consistent with previous research [9], the sample with signature 6 had a higher mutation rate.

We used the Database for Annotation, Visualization and Integrated Discovery [10] to identify altered pathways. The MAPK pathway was altered in 12 LSTs (85.7%), followed by the cAMP and Rap1 pathways (11 LSTs, 78.6%), and the Hippo pathway (10 LSTs, 71.4%) (Fig. S3).

Analysis of inactive mutations in different pathways (Fig. S3) indicated 12 inactive mutations in the Hippo/TGF‐β pathway. All mutated genes in this pathway harbored at least one inactive mutation (Fig. S4A and S4B). The upstream regulator *BMPR1A* (a receptor of bone morphogenetic proteins) contained 2 indels (p.P57fs and p.L209fs) and 1 variant at a splicing site (rs587781407) (Fig. S4A and S4B). The mutations in *TGFB2* (whose protein phosphorylates SMADs *via* TGF‐β receptors) were present in 2 LSTs (p.T23fs and p.P240Q) (Fig. S4A and S4B). Previous research reported that mutations of *ACVR1B* and *ACVR2A* (subunits of the membrane activin receptor complex in TGF‐β signaling) were common in CRC as well [8]. cAMP signaling contains the commonly mutated gene *GNAS*, which functions as an upstream factor that affects the generation of the cAMP, a universal second messenger (Fig. S4C and S4D). Mutations in *GLI1*, the final effector of the hedgehog signaling, and a transcriptional regulator that affects cellular behaviors in human cancers, were present in 2 LSTs (p.R268Q and p.L897fs) (Fig. S4C and S4D). Two mutations were detected in *SOX9* which affected Paneth cell differentiation and triggered the Wnt pathway [11].

Our WES data indicated that *APC* was the most frequently mutated gene, and its mutation was extensively investigated. We also found that the frequently mutated gene *GNAS* (a driver of CRC) had a mutational hotspot at codon 186, in which arginine was converted to histidine (p.R186H) or cysteine (p.R186C). The most frequent mutation in *GNAS* was at codon 201, and this mutation was reported in many types of adenocarcinomas [12]. The mutation at codon 186 had not been well characterized. We, therefore, performed validation of the *GNAS* p.R186C/R186H using Sanger sequencing. Among 5 of the 14 initial LSTs with *GNAS* p.R186C/R186H, 4 samples were successfully validated. Thirteen samples (16.5%) in the validation series of 79 LSTs had the *GNAS* p.R186C/R186H mutation, 11 with p.R186H, and 2 with p.R186C (Table S6; Figure 2A). All lesions with mutations were HGIN of the rectum. There were similar mutation frequencies in males and females (*P *> 0.05, Pearson's Chi‐squared test). The mutation frequency in HGIN did not differ significantly from LGIN (*P *> 0.05, corrected Pearson's Chi‐squared test). The frequency of *GNAS* p.R186C/R186H in the rectum was significantly higher than in the colon (*P *< 0.05, Pearson's Chi‐squared test). *GNAS* p.R186C/R186H also had a significant association with histological type (*P *< 0.05, Fisher's exact test), in that it was significantly more frequent in villous adenoma (VA) than tubular adenoma (TA) (*P *< 0.05, Pearson's Chi‐squared test). Lesions with this mutation were also significantly larger than wild‐type lesions (68.1 ± 34.5 mm vs. 36.8 ± 18.1 mm, *P *< 0.05, Welch *t*‐test; Figure 2B). However, among rectal tubulovillous adenoma (TVA) and VA, the *GNAS*‐mutated lesions were larger but without statistical difference (68.1 ± 34.5 mm vs. 49.8 ± 22.6 mm, *P *> 0.05, Welch *t*‐test; Figure 2C). None of the adjacent non‐tumor tissue samples carried the *GNAS* p.R186C/R186H mutation.

**FIGURE 2:**
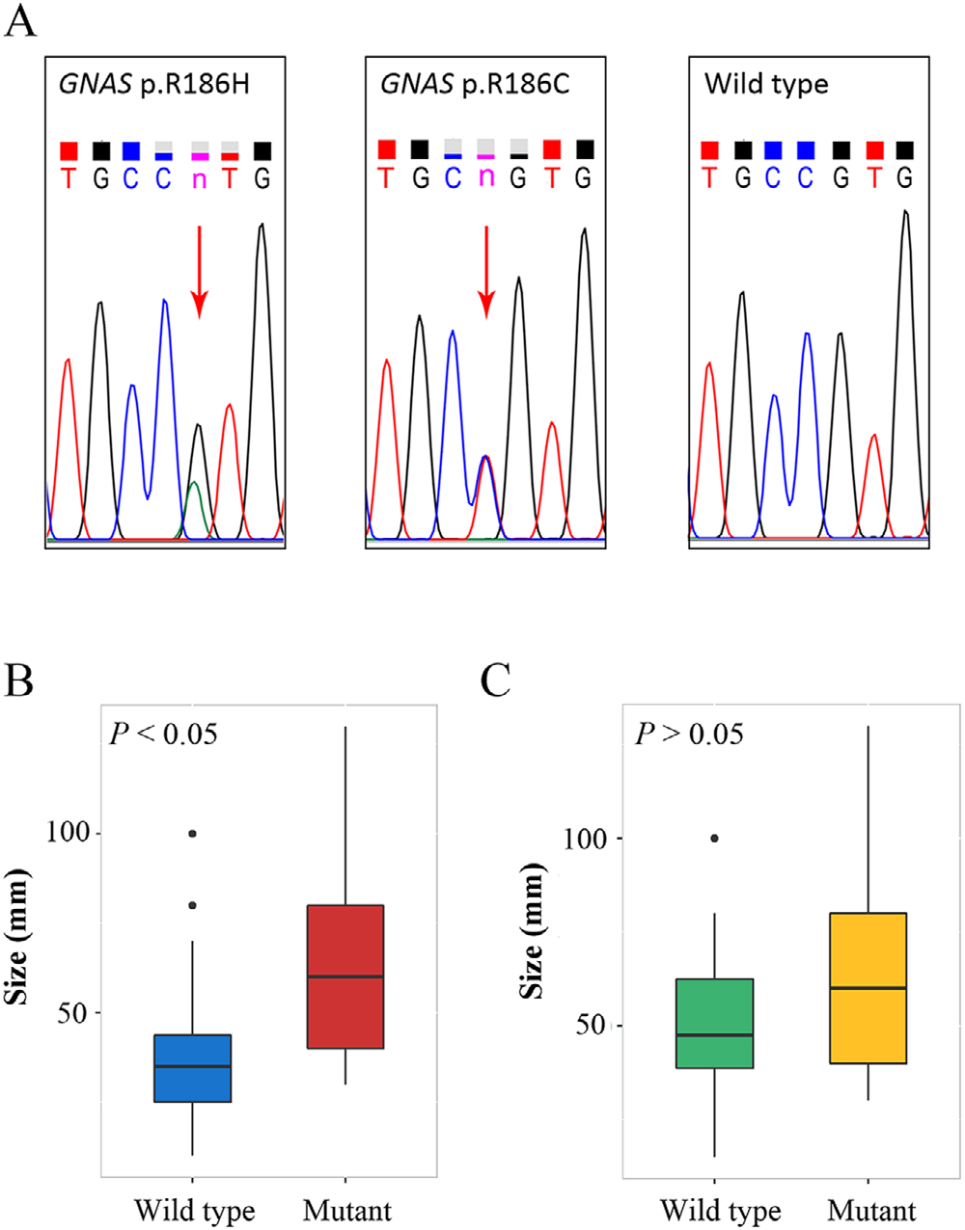
**Validation of *GNAS*** p.**R186C/R186H**. A. Sanger sequencing of *GNAS*. The sites with double peaks show a G > A substitution (p.R186H, left) or a C > T substitution (p.R186C, middle), whereas the wild‐type shows a single peak (right). B. Diameter of wild‐type and *GNAS*‐mutated lesions among all LSTs (*P *< 0.05, Welch *t*‐test). C. Diameter of wild‐type and *GNAS*‐mutated lesions in rectal TVA and VA (*P *> 0.05, Welch *t*‐test). *GNAS*: G protein subunit α, LST: laterally spreading tumor, TVA: tubulovillous adenoma, VA: villous adenoma

Existing data described that *GNAS* mutations are rare in CRC but relatively more frequent in precancerous lesions [8, 13], suggesting a role of this mutation during early tumorigenesis. Mutations at codons 201 and 186 are more frequent in villous adenomas [13]. Moreover, *GNAS* p.R186C/R186H was observed in LST but not colorectal adenoma, suggesting that it was unique to LST. Comparisons of different morphological types of CRN are needed to clarify the clinicopathological consequences of *GNAS* p.R186C/R186H. There is evidence that cAMP regulates cyclooxygenase‐2 (COX‐2) expression and affects colorectal tumorigenesis [14]. Another study reported an overexpression of COX‐2 in LST [15]. Therefore, it is possible that mutated *GNAS* affects LST development *via* the cAMP‐COX2 axis. More studies are needed to explore the functional implications of *GNAS* p.R186C/R186H.

To summarize, our WES on 14 LST with HGIN revealed 202 recurrently mutated genes, as well as 368 SMGs, a predominance of the mutational signature 1A, and the frequently altered Hippo/TGF‐β and cAMP/Rap1/MAPK pathways. Notably, we found a high frequency of *GNAS* p.R186C/R186H and an association of this mutation with tumor location (rectum) and pathological type (villous). Nonetheless, more samples are required to comprehensively elucidate the genomic landscape of LST. Our study provides a basis for understanding the molecular mechanism underlying the pathogenesis of LST, and may aid in the early diagnosis of CRC.

## AUTHORS’ CONTRIBUTIONS

SDL and AML contributed to obtain the funding, research design and project supervision; YYN contributed to experiment conduction, data analysis and manuscript writing; YRZ, YZ, LP, CJZ were responsible for sample collection; JSC and LH provided technical and material support. All authors read and approved the final manuscript.

## FUNDING

This research was mainly supported by grants from the National Nature Science Funds of China (grant no. 81772964), the Special Scientific Research Fund of Public Welfare Profession of National Health and Family Planning Commission (grant no. 201502026) and the Guangdong Gastrointestinal Disease Research Center (grant no. 2017B020209003).

## AVAILABILITY OF DATA AND MATERIALS

Methods and materials are available in supplementary information. Raw sequencing data have been deposited in NCBI database with BioProject ID: PRJNA529959.

## ETHICS APPROVAL AND CONSENT TO PARTICIPATE

The studies were conducted in accordance with Declaration of Helsinki. The sample collecting protocol was approved by the Ethics Committee of Nanfang Hospital (Permission No.: 2011‐094).

## CONSENT FOR PUBLICATION

Not applicable

## CONFLICT OF INTEREST

The authors declare that they have no conflicts of interest.

## Supporting information

Supplementary material 1: Methods and materials

Supplementary material 2: Supplementary tables and figures
