# Supplementary material for: Analysis of genetic alterations identifies the frequent mutation of GNAS in colorectal laterally spreading tumors
Source: Cancer Commun (Lond). 2020 Aug 6;40(11):636–40. doi: 10.1002/cac2.12085 (PMC7668477; doi:10.1002/cac2.12085)
Supplement: Supplementary file 1 — Supplementary material 1: Methods and materials [file CAC2-40-636-s002.pdf]

Methods and Materials

Sample collection and DNA extraction

Laterally spreading tumors (LSTs) along with paired adjacent non-tumor tissue samples were obtained from patients who had undergone endoscopic mucosal resection (EMR), endoscopic submucosal dissection (ESD) or colectomy at the Nanfang Hospital (Guangzhou, Guangdong, China) between May 1, 2016 and June 30, 2019. All subjects provided informed consent for the study participation. Patients with inflammatory bowel disease, familial adenomatous polyposis, melanosis coli or those who had preoperatively received radiotherapy or chemotherapy were excluded. Clinicopathological data were retrieved from the patients' medical records. For whole-exome sequencing (WES), formalin-fixed paraffin-embedded (FFPE) tissues were dissected into 10 μm-thick slices before they were treated with xylene substitute for 10 min and 100% alcohol for 2 min. Then, these tissues were micro-dissected using the Leica LMD6500 Microsystem (Leica Biosystyms, Wetzlar, Hessen, Germany) to separate high-grade intraepithelial neoplasia (HGIN) from the tumor and adjacent non-tumor tissues that were differentiated by histopathology. DNA was extracted using the QIAamp GeneRead DNA FFPE Kit (Qiagen, Hilden, Nordrhein-Westfalen, Germany) according to the manufacturer's instructions, and 200 ng genomic DNA [measured by Qubit® 2.0 Fluorometer (Thermo Fisher Scientific, Waltham, Massachusetts, USA) was used for the library preparation. Fresh tumor samples were divided into two parts, with one part being snap-frozen in −80 ℃ right after dissection and the other part being formalin-fixed for histopathological examination. DNA was extracted using the QIAamp DNeasy Blood and Tissue Kit (Qiagen) according to the manufacturer's instructions.

Histopathological evaluation of LST

Briefly, 4μm-thick tissue sections were cut and subjected to hematoxylin and eosin (H&E) staining according to standard procedures. Histological diagnosis was undertaken by 2 pathologists with over 5 years of working experience. The histological type of adenoma was determined based on the World Health Organization classifications (2010). Lesions with ≥ 75% villous component were classified as villous adenoma, lesions with 25%−75% villous component were classified as tubulovillous adenoma, lesions with ≤ 25% villous component were classified as tubular adenoma, and lesions with serrated structure were classified as serrated adenoma. Dysplasia was classified as HGIN (including intramucosal carcinoma) or low-grade intraepithelial neoplasia (LGIN) according to the Vienna Criteria[1]. Submucosal carcinoma was characterized as lesions invading into the submucosa layer or even deeper layers.

Whole-exome Sequencing and Mutation Calling

WES was conducted by the Shanghai Biotechnology Corporation (Shanghai, China). Briefly, whole-exome capture was carried out using Agilent's SureSelect Human All Exon V6Kit (58Mb, Agilent Technologies, Palo Alto, CA, USA). For each sample to be sequenced, individual library preparation, hybridization, and capture were carried out according to the protocol of SureSelect^{XT} Target Enrichment System for Illumina Paired-End Sequencing Library (Agilent Technologies). The 2100 Bioanalyzer High Sensitivity DNA Assay (Agilent Technologies) was used to assess quality and size range as instructed in the reagent kit guide. Sequencing was undertaken on an Illumina HiSeq X instrument (Illumina Inc., San Diego, CA, USA) according to the manufacturer's protocol for paired-end 2×150 bp reads. FastQ

files were evaluated by the FastQC and statistics including number of total reads and percentage of bases with average quality of Q ≥ 20 (Q20, as %) were calculated. Paired-end reads were aligned to the reference genome from the University of California, Santa Cruz (UCSC) hg19 using the Burrows-Wheeler Alignment (BWA) tool [2]. BAM files were preprocessed using Genome Analysis Toolkit [3, 4]; thereafter, the somatic nucleotide variant (SNV) and insertions/deletions (indels) were called by the MuTect2 algorithm[5]. We used the ANNOVAR software[6] to annotate these somatic mutations. Mutation burden was calculated as the number of somatic mutations/total covered bases. Mutation sites with reliability assessment of “pass” identified using the VariantRecalibrator and ApplyRecalibration tools would be submitted for subsequent analysis.

Mutational signature analysis

Different combinations of mutation types generated by different mutational processes were termed as mutational signatures. Taking into account 6 mutational substitutions (C>A, C>G, C>T, T>A, T>C, T>G) and their immediate 5’and 3’ bases, there were 96 possible trinucleotides. We used the deconstructSigs package[7] in R software[8] to obtain the mutational signature of each paired sample on the basis of the fraction of mutations found in the context of each of the 96 trinucleotides.

Determination and integrative analysis of significantly mutated genes (SMGs)

Sites annotated as “unknown” and “synonymous SNV” were discarded. Here, we locally ran the MutSigCV algorithm [9] and defined genes with P values < 0.05 as SMGs which referred to mutated genes with higher mutation frequency than the background mutation rate. To identify the known driver genes, we compared sample SMGs with known driver mutations

using the DriverDB database (http://driverdb.tms.cmu.edu.tw/driverdbv2/). The Database for Annotation, Visualization and Integrated Discovery (v6.8) [10] was used to map SMGs to the Kyoto Encyclopedia of Genes and Genomes database to analyze the pathway alterations in LST. We considered the pathway “altered” if a mutated gene was involved in it.

Sanger Sequencing

Based on the results of WES, to validate the G protein subunit α (*GNAS*) codon 186 mutation, Sanger sequencing was conducted in 5 of the 14 initial cases with this mutation and another cohort of 79 LSTs. The forward and reverse primers for *GNAS* were 5´-AACTGTGGGACGGTCACTTC-3´ and 5´-CACCCACGTCAAACATGCTG-3´, respectively. DNA product was purified after 35 cycles of polymerase chain reaction (PCR). Sanger sequencing was carried out by the Beijing Ruibiotech Corporation (Beijing, China).

Statistical analysis and generation of high-quality graphs

Statistical analysis was conducted and statistical graphs were generated using the R software (v3.6.1)[8]. The oncoPrints were generated using the ComplexHeatmap package (v2.0.0)[11]. Lolliplots of adenomatous polyposis coli (*APC*) and *GNAS* were generated using the cBioPortal online tool (cbioportal.org).

References

1. Dixon MF. Gastrointestinal epithelial neoplasia: Vienna revisited. Gut. 2002;51(1):130-1.
2. Li H, Durbin R. Fast and accurate short read alignment with Burrows-Wheeler transform. Bioinformatics. 2009;25(14):1754-60.
3. DePristo MA, Banks E, Poplin R, Garimella KV, Maguire JR, Hartl C, et al. A framework for variation discovery and genotyping using next-generation DNA sequencing data. Nat Genet. 2011;43(5):491-8.
4. McKenna A, Hanna M, Banks E, Sivachenko A, Cibulskis K, Kernytsky A, et al. The Genome Analysis Toolkit: a MapReduce framework for analyzing next-generation DNA sequencing data. Genome Res. 2010;20(9):1297-303.
5. Cibulskis K, Lawrence MS, Carter SL, Sivachenko A, Jaffe D, Sougnez C, et al. Sensitive detection of somatic point mutations in impure and heterogeneous cancer samples. Nature biotechnology. 2013;31(3):213-9.
6. Wang K, Li M, Hakonarson H. ANNOVAR: functional annotation of genetic variants from high-throughput sequencing data. Nucleic Acids Res. 2010;38(16):e164.
7. Rosenthal R, McGranahan N, Herrero J, Taylor BS, Swanton C. DeconstructSigs: delineating mutational processes in single tumors distinguishes DNA repair deficiencies and patterns of carcinoma evolution. Genome Biol. 2016;17(1):31.
8. Hackenberger BK. R software: unfriendly but probably the best. Croatian medical journal. 2020;61(1):66-68.
9. Lawrence MS, Stojanov P, Polak P, Kryukov GV, Cibulskis K, Sivachenko A, et al. Mutational heterogeneity in cancer and the search for new cancer-associated genes. Nature. 2013;499(7457):214-18.
10. Huang da W, Sherman BT, Lempicki RA. Bioinformatics enrichment tools: paths toward the comprehensive functional analysis of large gene lists. Nucleic Acids Res. 2009;37(1):1-13.
11. Gu Z, Eils R, Schlesner M. Complex heatmaps reveal patterns and correlations in multidimensional genomic data. Bioinformatics. 2016;32(18):2847-9.
