# Supplementary material for: Analysis of genetic alterations identifies the frequent mutation of GNAS in colorectal laterally spreading tumors
Source: Cancer Commun (Lond). 2020 Aug 6;40(11):636–40. doi: 10.1002/cac2.12085 (PMC7668477; doi:10.1002/cac2.12085)
Supplement: Supplementary file 2 — Supplementary material 2: Supplementary tables and figures [file CAC2-40-636-s001.pdf]

Supplementary Tables and Figures

Supplementary Tables

Table S1. Demographic and clinicopathological data of the 14 LSTs used for WES

Patient ID	Gender	Age (years)	Histological classification	Vienna classification	Size (mm)	Tumor location
P01	F	40	TVA	HGIN	80	Rectum
P02	F	63	TVA	HGIN	50	Rectum
P03	M	63	TA	HGIN	25	Ascending colon
P04	F	54	VA	HGIN	60	Transverse colon
P05	F	50	VA	HGIN	25	Rectum
P06	M	57	TA	HGIN	40	Sigmoid colon
P07	M	52	TA	HGIN	30	Rectum
P08	M	54	VA	HGIN	110	Rectum
P09	M	73	VA	HGIN	60	Rectum
P10	F	71	TVA	HGIN	25	Transverse colon
P11	M	49	TVA/CA	HGIN	20	Transverse colon
P12	F	49	TVA	HGIN	30	Cecum
P13	F	58	VA	HGIN	30	Rectum
P14	M	43	TA	HGIN	16	Rectum

LSTs: laterally spreading tumors, WES: whole-exome sequencing, F: female, M: male, TVA: tubulovillous adenoma, VA: villous adenoma, TA: tubular adenoma, CA: cancer, HGIN: high-grade intraepithelial neoplasia.

Table S2. Number of mutations and mutation burden of each sample

Patient ID	Number of SNVs	Number of indels	Number of mutated genes	Mutation burden (per Mb)
P01	202	24	90	3.85
P02	204	17	95	3.76
P03	334	83	198	7.10
P04	149	26	79	2.98
P05	130	12	65	2.42
P06	2437	535	1090	50.62
P07	313	33	151	5.89
P08	213	42	115	4.34
P09	147	78	96	3.83
P10	155	34	81	3.22
P11	233	23	200	4.36
P12	161	29	91	3.24
P13	165	33	86	3.37
P14	133	17	62	2.55

SNVs: somatic nucleotide variants, indels: insertions/deletions.

Table S3. Number of mutations in different classifications

Variant classification	Number of mutations per sample													
	P01	P02	P03	P04	P05	P06	P07	P08	P09	P10	P11	P12	P13	P14
Missense SNV	74	79	137	65	54	983	133	93	61	55	100	68	61	43
Nonsense SNV	7	8	11	3	3	57	7	4	10	4	2	8	6	6
Indel	12	7	44	11	8	109	13	20	32	25	16	18	20	12
Splicing site	0	2	19	0	0	10	1	1	3	3	6	4	6	2

SNV: somatic nucleotide variant, indel: insertion/deletion.

Table S4. The 368 significantly mutated genes identified by MutSigCV algorithm

AASS	CBFB	EMX2	JARID2	OR5P3	RNF103	TEAD2
ABCD3	CCDC36	EXTL2	JPH2	OR6X1	RNF185	TECR
ACADVL	CCDC63	FAM118B	KANK4	OTUD7B	ROPN1B	TGFB2
ACO2	CCDC67	FAM19A5	KCND2	OXT	ROS1	TGIF1
ACSF2	CCIN	FAM50B	KCNJ4	PAK4	RPL11	THRB
ACVR1B	CCR2	FASN	KCNMB1	PALB2	RPL23A	TM4SF4
ACVR2A	CD151	FBXW7	KCTD20	PAM	RPN2	TM7SF2
ADAM30	CD28	FGG	KIAA1147	PAPSS1	RPUSD1	TMEM147
ADD3	CD6	FLCN	KIF23	PAX3	RRAS	TMEM190
AHCYL1	CDA	FLVCR2	KIF3C	PAX7	RSAD2	TMEM42
AKR1B1	CDC7	FMR1NB	KPNA4	PCBP1	RSPH4A	TMEM44
AKR1B15	CDH10	FSD2	KRAS	PCCB	RSPO1	TMEM51
ALDH1L1	CDH9	FTSJ3	KRT10	PCDHGA6	RUNX2	TMEM82
ALDH1L2	CDHR2	G0S2	LDLRAD1	PDC	SAMD3	TMEM8C
ALOX12B	CENPN	GABPB2	LEPROT	PER2	SARDH	TMPRSS11A
AMBP	CKAP4	GBP4	LPAR2	PEX11B	SDK1	TNFRSF10A
ANAPC5	CLASP2	GBX1	LPCAT3	PGAM4	SERPINE3	TRAF2
ANKRD35	CLEC7A	GDI2	LRGUK	PHACTR1	SF3B3	TRAM1L1
ANKS4B	CLEC9A	GIPC1	LRPAP1	PHLDA1	SGSM1	TRAPPC2L
ANO5	CLK1	GLB1L3	LZIC	PHLDB3	SH2D6	TRNT1
AP1AR	CLPTM1L	GLI1	MAN2A1	PIAS3	SHROOM2	TRPC3
APBB2	CNBD1	GNAS	MARCH2	PIGR	SKA3	TRPC7
APC	CNGA1	GNAT3	MARVELD2	PIRT	SLAIN2	TSEN34
AQR	CPA1	GNPAT	MATR3	PLEKHB2	SLC17A4	TTK
ARHGAP22	CREG2	GNPDA1	MCM3	PLXDC2	SLC20A2	TUBGCP4
ARID3A	CROT	GNS	MED28	PNPO	SLC25A14	TUSC5
ARID5B	CRYBA1	GPR171	MED30	POLG	SLC25A3	UBASH3B
ARL4D	CSN3	GPR82	MFF	PPHLN1	SLC30A5	UBE2E2
ARNT	CTDP1	GRAMD1C	MRPS34	PRG4	SLC8A3	UBQLN2
ARSJ	CTLA4	GRK7	MS4A13	PRR5	SMAD7	UCP2
ART3	CTNNA1	GSTA3	MSI1	PRRG1	SMCP	UROD
ASXL2	CXorf30	GSTM3	MTMR2	PRSS21	SMG7	USP38
ATP13A3	CYB5R4	GSTP1	MYT1L	PRSS3	SMPD3	USP42
ATP1B1	CYLC1	GTF2A1	NCAM2	PSD	SNAP25	USP6
ATP2B3	CYP11B1	H2BFWT	NDFIP1	PSKH2	SOX9	UTP14A
AXIN2	CYP1B1	HAPLN4	NEDD4L	PSMB1	SPACA1	VCP
BAX	CYTH4	HIAT1	NENF	PSMB6	SPARCL1	WDFY2
BCL10	DAPL1	HIST1H1A	NEUROG3	PTPN4	SPATA9	WDR44
BCL9	DDAH1	HIST1H2BA	NEXN	PWP1	SPN	WDR89
BET1L	DDX20	HIST1H2BD	NFIX	PWWP2A	SRRM1	XPO7
BMPR1A	DEPDC7	HIST1H4E	NFKBIZ	RAB4A	SRRM4	ZFP36L2
BRAF	DIRC1	HIST1H4G	NKAIN4	RAC1	STIL	ZHX2
BRI3	DLEC1	HMGXB3	NLGN3	RAF1	STXBP6	ZIC3

C11orf57	DLG3	HPS1	NOSIP	RBM19	SULT2A1	ZMAT1
C15orf62	DPY19L2	HRAS	NOVA1	RBM6	SULT6B1	ZMPSTE24
C19orf71	DRD3	HS6ST1	NRAS	RBP4	SYAP1	ZNF131
C1QA	DSG1	ICAM2	OBP2A	RCAN2	SYNGR2	ZNF185
C2orf71	DSG4	IFNA5	OCIAD1	RGMB	SYPL1	ZNF19
C5orf49	DUSP26	IFNGR2	OR1J2	RHAG	SYT7	ZNF208
C6orf89	EGFL7	IGLON5	OR2AT4	RHOA	TAC4	ZNF395
C9orf89	EIF3A	IRX5	OR4D5	RLTPR	TCF20	ZNF750
CACNA1B	EIF6	ITCH	OR52D1	RND3	TCTN3	ZNHIT1
CACNG4	ELOVL5	ITPKB	ZNHIT6			

Table S5. Mutations of driver genes among the significantly mutated genes

Gene	Patient ID	Chromosome	Protein change	AF	Variant classification	dbSNP-RS
ACVR2A	P06	chr2	p.K327fs	0.346	indel	rs764719749
APC	P12	chr5	p.G617fs	0.250	indel	-
APC	P10	chr5	p.E745X	0.618	nonsense SNV	-
APC	P01	chr5	p.W535X	0.514	nonsense SNV	rs398123116
APC	P14	chr5	p.Q1360X	0.596	nonsense SNV	rs121913329
APC	P06	chr5	p.R223X	0.311	nonsense SNV	rs587781392
APC	P12	chr5	p.S1527X	0.400	nonsense SNV	-
APC	P05	chr5	p.G1394fs	0.354	indel	-
APC	P06	chr5	p.R232X	0.340	nonsense SNV	rs397515734
APC	P01	chr5	p.H1472fs	0.357	indel	rs398123122
APC	P09	chr5	p.E1291X	0.421	nonsense SNV	-
APC	P03	chr5	p.R284X	0.381	nonsense SNV	rs137854568
AXIN2	P12	chr17	p.Y138C	0.449	missense SNV	-
AXIN2	P06	chr17	p.G665fs	0.370	indel	rs267606674
BCL9	P10	chr1	p.P514fs	0.128	indel	rs781994033
BRAF	P13	chr7	p.V600E	0.357	missense SNV	rs113488022
BRAF	P06	chr7	p.P403fs	0.127	indel	rs777474487
BRAF	P05	chr7	p.D594N	0.367	missense SNV	rs397516896
CACNA1B	P06	chr9	p.S1237L	0.437	missense SNV	rs754545679
CACNA1B	P08	chr9	p.I49F	0.203	missense SNV	-
CACNA1B	P07	chr9	p.A1336T	0.291	missense SNV	-
CDH10	P04	chr5	p.S569X	0.442	nonsense SNV	-
CDH10	P10	chr5	p.L480F	0.039	missense SNV	rs77909063
CDH9	P01	chr5	p.A38T	0.407	missense SNV	-
CTNNA1	P03	chr5	p.L61fs	0.079	indel	-
CYP11B1	P06	chr8	p.A348T	0.412	missense SNV	rs6407
CYP11B1	P11	chr8	p.V103L	0.223	missense SNV	rs775858610
FBXW7	P03	chr4	p.R361L	0.214	missense SNV	-
FBXW7	P01	chr4	p.R347C	0.333	missense SNV	rs867384286
FBXW7	P03	chr4	p.R104X	0.319	nonsense SNV	-
FBXW7	P05	chr4	p.R347C	0.379	missense SNV	rs867384286
FBXW7	P09	chr4	p.R347C	0.400	missense SNV	rs867384286
GNAS	P01	chr20	p.R186H	0.450	missense SNV	rs121913495
GNAS	P06	chr20	p.D583N	0.024	missense SNV	
GNAS	P02	chr20	p.R186H	0.426	missense SNV	rs121913495
GNAS	P13	chr20	p.R186C	0.527	missense SNV	rs11554273
GNAS	P09	chr20	p.R186H	0.486	missense SNV	rs121913495
GNAS	P11	chr20	p.P53L	0.012	missense SNV	rs763257494
GNAS	P08	chr20	p.R186C	0.447	missense SNV	rs11554273
HS6ST1	P09	chr2	p.R185X	0.015	nonsense SNV	rs764216851
ITPKB	P06	chr1	p.E207fs	0.190	indel	-

ITPKB	P07	chr1	p.R760Q	0.033	missense SNV	rs200031424
KCND2	P06	chr7	p.R293X	0.091	nonsense SNV	-
KRAS	P02	chr12	p.G12D	0.393	missense SNV	rs121913529
KRAS	P01	chr12	p.G13D	0.550	missense SNV	rs112445441
KRAS	P08	chr12	p.G13D	0.316	missense SNV	rs112445441
MAN2A1	P01	chr5	p.R499X	0.053	nonsense SNV	-
NRAS	P12	chr1	p.Q61K	0.311	missense SNV	rs121913254
NRAS	P09	chr1	p.G13R	0.125	missense SNV	rs121434595
PCBP1	P03	chr2	p.L100Q	0.256	missense SNV	-
PCBP1	P06	chr2	p.G116C	0.281	missense SNV	-
POLG	P06	chr15	p.T690M	0.313	missense SNV	rs201677865
RAC1	P06	chr7	p.R68C	0.087	missense SNV	-
RAF1	P03	chr3	*	0.094	indel	-
RUNX2	P09	chr6	p.V207fs	0.094	indel	-
RUNX2	P03	chr6	p.R231S	0.034	missense SNV	-
RUNX2	P09	chr6	p.V207fs	0.094	indel	-
SDK1	P05	chr7	p.Y261H	0.439	missense SNV	-
SDK1	P11	chr7	p.E663D	0.067	missense SNV	-
SDK1	P07	chr7	p.F106L	0.156	missense SNV	-
SDK1	P14	chr7	p.T592M	0.545	missense SNV	rs368048544
SDK1	P04	chr7	p.R369W	0.164	missense SNV	rs780003658
SOX9	P01	chr17	p.F294fs	0.447	indel	-
SOX9	P12	chr17	p.Q393X	0.477	nonsense SNV	-
TGIF1	P03	chr18	p.Y62_N63delinsX	0.757	indel	-
TGIF1	P05	chr18	p.L80fs	0.393	indel	-
TGIF1	P02	chr18	p.G117fs	0.451	indel	-
USP42	P06	chr7	p.K1234fs	0.343	indel	rs759165525
USP42	P04	chr7	p.G453R	0.380	missense SNV	rs371602141
USP6	P10	chr17	p.R1249fs	0.160	indel	-
ZFP36L2	P12	chr2	p.R160fs	0.311	indel	-

SNV: somatic nucleotide variant, indel: insertion/deletion, AF: allele frequency.

dbSNP-RS: The identify number in NCBI dbSNP database

* p.S29_P30delinsFDDQQWFWIQRCLVX

Table S6. Demographic and clinicopathological characteristics of samples used for validation by Sanger sequencing

Classification		Total [*n* (%)]	Mutant [*n* (%)]
Histological classification	TA	21 (26.6)	0 (0)
	VA	34 (43.0)	10 (29.4)
	TVA	22 (27.8)	3 (13.6)
	SA	2 (2.5)	0 (0)
Vienna classification	LGIN	15 (19.0)	0 (0)
	HGIN	64 (81.0)	13 (20.3)
Tumor location	Rectum	44 (55.7)	13 (29.5)
	Sigmoid colon	6 (7.6)	0 (0)
	Descending colon	3 (3.8)	0 (0)
	Transverse colon	6 (7.6)	0 (0)
	Ascending colon	11 (13.9)	0 (0)
	Cecum	9 (11.4)	0 (0)
Gender	M∶F	35:44	5:8
Age（yrs）	Mean (range)	61 (22−83)	60 (22−80)
Tumor size（mm）	Mean (±SD)	42.0 (±24.3)	68.1 (±34.6)

F: female, M: male, TVA: tubulovillous adenoma, VA: villous adenoma, TA: tubular adenoma, SA: serrated adenoma, HGIN: high-grade intraepithelial neoplasia, LGIN: low-grade intraepithelial neoplasia, SD: standard deviation.

Supplementary Figures

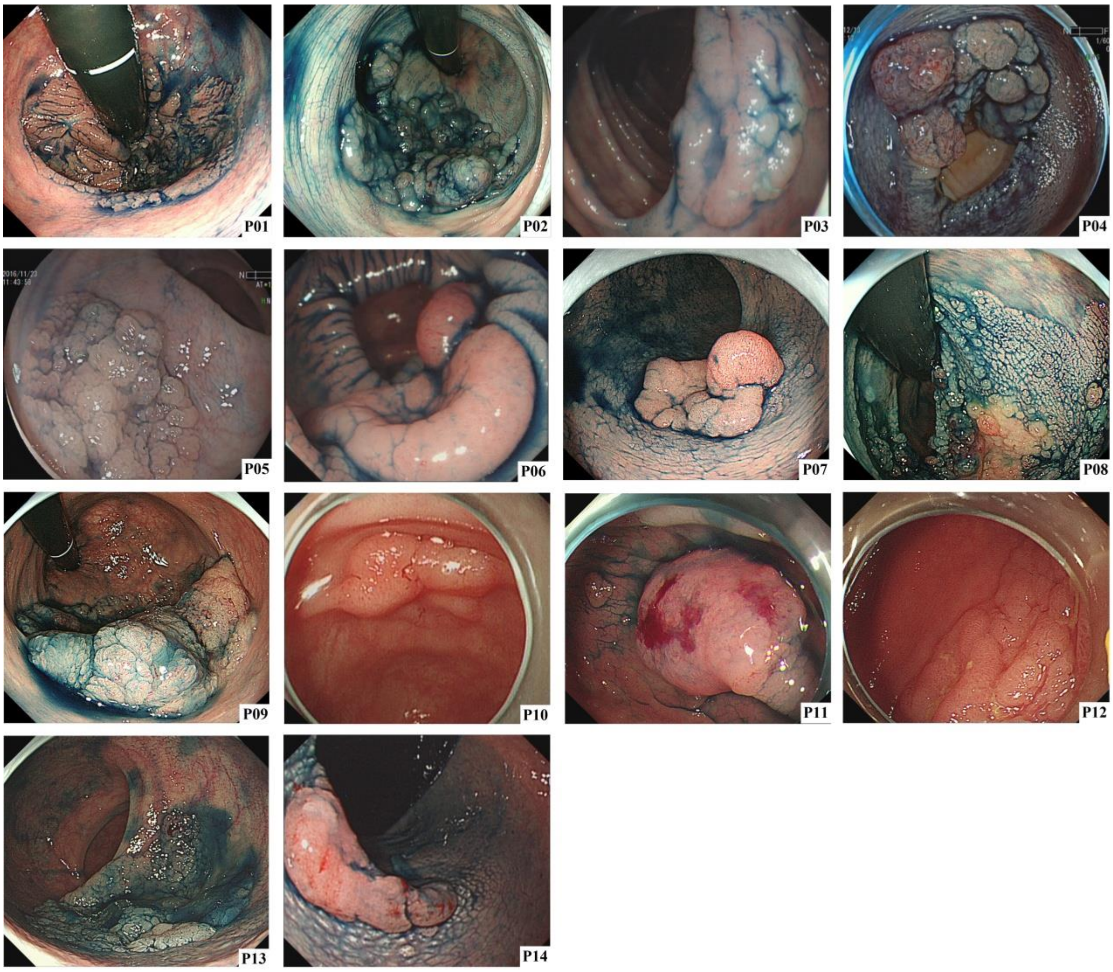

Figure S1. The 14 LSTs diagnosed by colonoscopy for WES

The indigo carmine staining was applied. The non-tumor tissue of case P14 was obtained more than 10 cm away from the tumor. LSTs: laterally spreading tumors, WES: whole-exome sequencing.

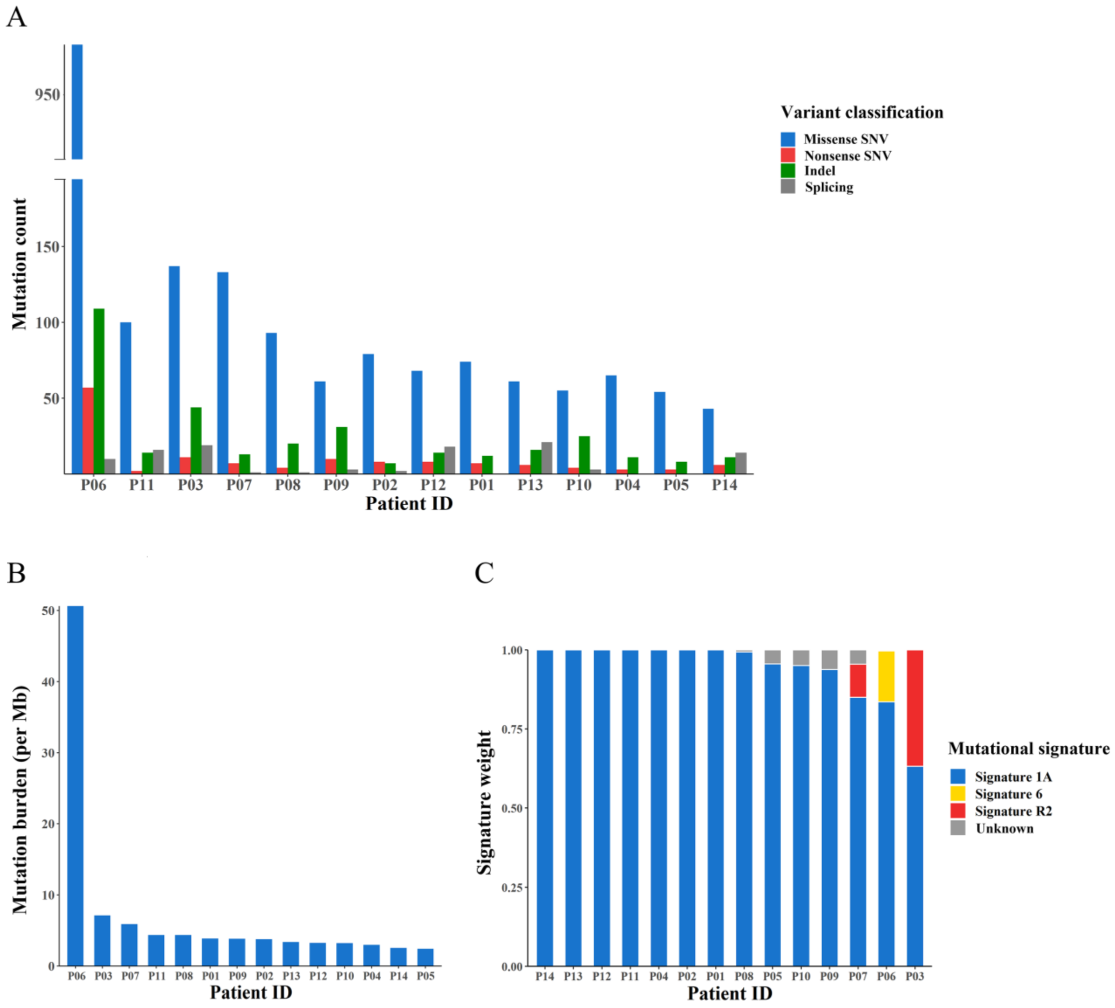

Figure S2. The number of mutations, mutation burden, and signature of LST

A. The number of mutations identified by the MuTect2 algorithm. The mutations were classified as missense SNV, nonsense SNV, indel, and variant at the splicing site. B. Mutation burden of LST. The mutation burden was calculated as the number of somatic mutations/total covered bases. C. Mutation signatures uncovered using the deconstructSigs package. Mutational signatures 1A, 6, and R2 were extracted in LST. LST: laterally spreading tumor, SNV: somatic nucleotide variant, indel: insertion/deletion.

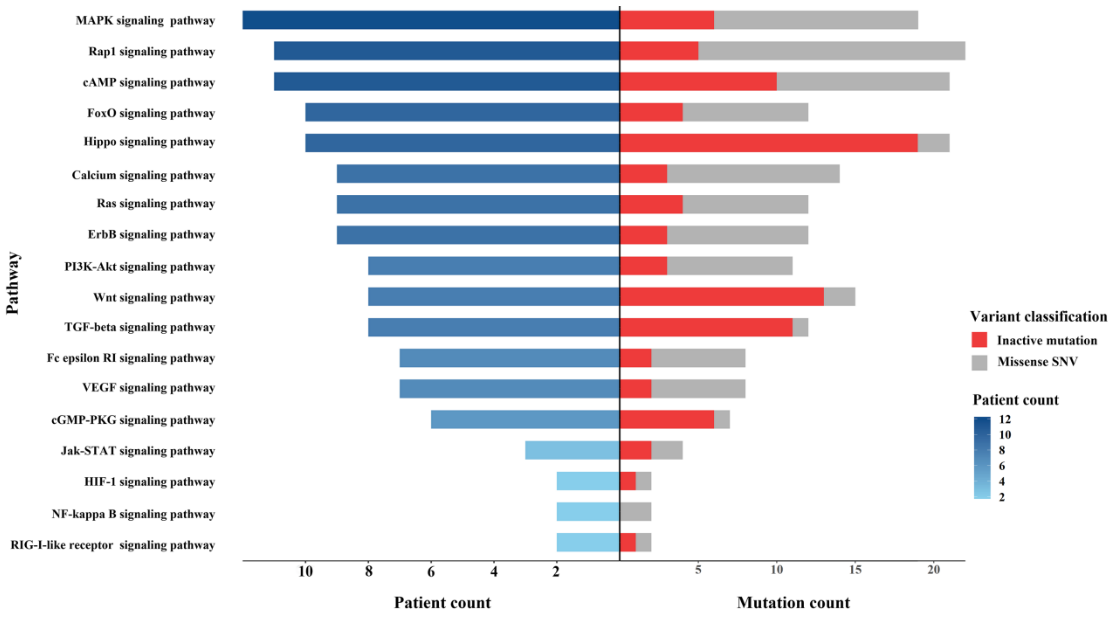

Figure S3. Altered pathways in LST

The bar chart shows the frequencies of alterations in cancer-related signaling pathways. The bars on the left side show the prevalence of each altered pathway. The bars on the right side show the number of mutations by classification of missense SNV and inactive mutation. Inactive mutation was defined as a nonsense SNV, indel, or variant at the splicing site. LST: laterally spreading tumor, SNV: somatic nucleotide variant, indel: insertion/deletion.

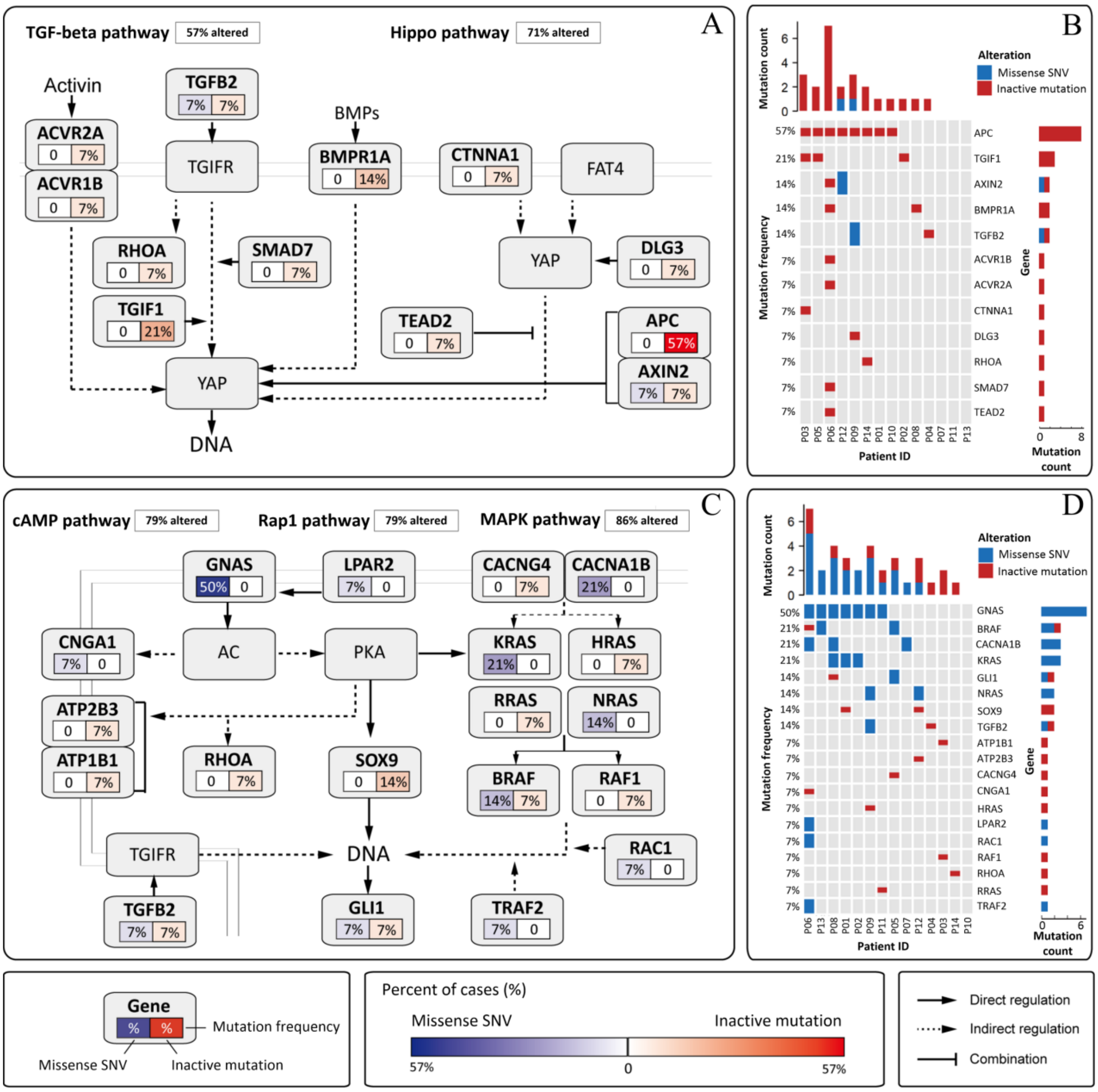

Figure S4. Genetic changes of the Hippo/TGF-β and cAMP/Rap1/MAPK signaling pathways

A. The frequencies of genetic alterations in the Hippo/TGF-β signaling pathway based on the KEGG database. Mutation frequency is expressed as a percentage of cases; missense SNVs are shown in blue and inactive mutations in red. B. OncoPrint for the mutated genes in the Hippo/TGF-β signaling pathway. C. The frequencies of genetic alterations in the cAMP/Rap1/MAPK signaling pathway based on the KEGG database. Mutation frequency is expressed as percentage of cases; missense SNVs are shown in blue and inactive mutations in red. D. OncoPrint for the mutated genes in the cAMP/Rap1/MAPK signaling pathway. Alterations were classified as missense SNV and inactive mutation which was defined as a nonsense SNV, indel, or variant at splicing sites. TGF-β: transforming growth factor-β, cAMP: cyclic adenosine monophosphate, Rap1: ras- proximity 1, MAPK: mitogen-activated protein

kinase, KEGG: the Kyoto Encyclopedia of Genes and Genomes, SNV: somatic nucleotide variant, indel: insertion/deletion.
